# How Much Do Side Effects Contribute to Discontinuation? A Longitudinal Study of IUD and Implant Users in Senegal

**DOI:** 10.3389/fgwh.2021.804135

**Published:** 2022-01-28

**Authors:** Dawn Chin-Quee, Mohamed Diadhiou, Margaret Eichleay, Ahmed Youssef, Mario Chen, Alissa Bernholc, John Stanback

**Affiliations:** ^1^Family Health International 360, Durham, NC, United States; ^2^Centre Régional de Formation, de Recherche et de Plaidoyer en Santé de la Reproduction (CEFOREP), Dakar, Senegal

**Keywords:** long-acting reversible contraception, implant, intrauterine device, longitudinal, Senegal

## Abstract

**Introduction:**

In Senegal, discontinuation due to sides effects of long-acting, reversible contraceptives (LARCs) is relatively low; 5% of new implant acceptors and 11% of new IUD acceptors stop using in their first year because of health or side effect concerns. This study investigated factors associated with LARC discontinuation in the first 12 months of use in Senegal and explored how LARC users cope with side effects.

**Methods:**

This mixed-method study involved quantitative interviews at five time points with LARC acceptors recruited from three service channels between February 2018 and March 2019. Qualitative interviews were conducted in August 2018 with a subset of those who experienced side effects. Logistic regression models identified factors associated with discontinuation due to side effects and discontinuation for any reason. Twelve-month discontinuation rates due to side effects were also estimated using a cumulative incidence function (CIF) approach to account for time to discontinuation.

**Results:**

In logistic models, method choice (IUD or implant) [OR = 3.15 (95% CI: 1.91–5.22)] and parity [OR = 0.81 (95% CI: 0.7–0.94)] were associated with discontinuation due to side effects; IUD users and women with fewer children were more likely to discontinue. Results for all-cause discontinuation were similar: method choice [OR = 2.39 (95% CI: 1.6–3.58)] and parity [OR = 0.86 (95% CI: 0.77–0.96)] were significant predictors. The 12-month side effect CIF discontinuation rate was 11.2% (95% CI: 7.9–15.0%) for IUDs and 4.9% (95% CI: 3.5–6.6%) for implants. Side effect experiences varied, but most women considered menstrual changes the least acceptable. No statistically significant differences across services channels were observed.

**Conclusions:**

In this study in Senegal, the choice between implants and IUDs had a significant impact on continuation, and women with more children continued LARC methods longer, despite side effects.

## Introduction

Long-acting, reversible contraceptives (LARCs) including sub-dermal implants and intrauterine devices (IUDs) are increasingly used for voluntary family planning (FP) in Africa, with implants, in particular, seeing marked increases in users. For instance, in the past decade, implants have become the most popular contraceptive methods in four countries: Senegal, Burkina Faso, Kenya and Ghana (1, 2). Data from service delivery organizations such as Marie Stopes International (MSI) show a similar trend, with steep upswings in voluntary implant uptake over the last few years, as implants became more readily available (3). The copper IUD is less popular than implants in sub-Saharan Africa, but because it is a longer-acting, highly effective method, low- and middle-income countries have promoted its voluntary uptake and use (4).

While discontinuation of LARCs is not nearly as high as that for short acting contraceptive methods, it nonetheless occurs and can leave women vulnerable to pregnancy if they do not adopt another method. According to a Demographic and Health Survey (DHS) analytical study in low-income countries, 9% of women discontinue implants and 15% discontinue IUDs within the first year of use (5). Other studies have looked at varying timeframes for discontinuation—from six months to as long as 24 months, with intervals shorter than 12 months expected to provide insight into women's early decisions to discontinue. Sznajder et al. looking at both 6 and 12-month discontinuation rates of IUD and implant users, found combined 11.3 and 21.9% 6 and 12-month discontinuation rates, respectively (6). The most common reason cited for discontinuation among IUD users was expulsion, and among implant users, bleeding. In the well-known Contraceptive CHOICE Project, comparison of LARC and non-LARC methods over a 24-month period revealed an overall continuation rate of 77% for LARC methods (levonorgestrel intrauterine system, copper IUD, implant) and 41% for non-LARC methods (pill, patch, ring) in an urban population in the United States (7). Reasons for discontinuation were not provided, but women who were Black (vs. race other than Black or White), under 20 years old and with a prior history of sexually transmitted infections were at higher risk for discontinuation (8).

Studies identify a range of factors that contribute to LARC discontinuation (9), particularly side effects. Both the copper IUD and implant share irregular bleeding, abdominal pain and infections as potential side effects, but the hormones in implants may also cause breast pain, headaches, moodiness, nausea/vomiting, weight gain and lack of sexual desire. Side effects specific to IUDs include pain with sex and expulsions. The DHS analytical study mentioned earlier found that four in ten episodes of LARC discontinuation were attributed to side effects or health concerns, more than double that of other modern methods combined (5). Research by Bahamondes et al. shows that good-quality counseling, defined as “appropriate counseling about effectiveness, safety, side effects and benefits of all methods available…,” motivates women to persevere and manage side effects (10). Even more encouraging, this counseling can be provided routinely, rather than through intensive methods as demonstrated in a 2014 study that found no differences in discontinuation rates between women who received routine counseling vs. intensive counseling (11).

There is a dearth of information—specifically from the client's point of view—on how to help women effectively manage side effects associated with LARCs and on how service delivery factors may affect a decision to continue or discontinue the method. A scoping review of contraceptive-induced menstrual bleeding changes underscored this gap and noted that most studies examining reasons for non-use, method dissatisfaction and discontinuation were conducted in developed, high-income countries (12). Other social, cultural, and attitudinal factors that influence experiences with side effects also warrant attention, as described in a Population Council report that identified gaps in our knowledge about how women (and men) “interpret side effects… that lead to discontinuation, and what motivates them to continue or discontinue when experiencing them” (13). Enhanced understanding of these factors and their interplay is important, as it could inform adjustments to service delivery guidelines and approaches.

Senegal is a West African country with about 16 million inhabitants, half of whom are under 18, a high total fertility rate (4.7 children per woman), and relatively high unmet need for contraception (22%) (1). From 2012 to 2019, contraceptive use among women in union increased substantially from 16 to 27%, and LARCs played an important role in this increase with implant use increasing from 3 to 10%, supplanting injectables as the most common contraceptive method in Senegal (1). Injectables remain popular, at 8%, followed by oral contraceptive pills (4%) and IUDs (2%) (1). Side effects are a common cause for discontinuation; 31.5% of discontinuers of all methods cited side effects as the primary reason (14). Additionally, while the 12-month all-cause discontinuation rates of pills and injectables were 40 and 39%, respectively, only 11% of implant users discontinued in that same time period (14). In Senegal, contraceptive methods are provided through the public and private sectors. In 2019, 9% of women in Senegal obtained their method of family planning in the private sector while 90% obtained it in the public sector including static health facilities (88%) and alternatives like mobile clinics (2%) (1, 14).

Collaborating with the Senegalese Ministry of Health, FHI 360, the Centre Régional de Formation, de Recherche et de Plaidoyer en Santé de la Reproduction (CEFOREP) and Marie Stopes International (MSI) conducted a study to build on our existing knowledge of LARC acceptance and use. The primary objective of the study was to understand the factors, including side effects that are associated with LARC continuation and discontinuation in the first twelve months of use. The secondary objective was to explore how LARC users cope with side effects.

## Materials and Methods

### Study Design and Participants

We conducted a mixed-method longitudinal study in four regions of Senegal: Dakar, Thiès, Kaffrine and Diourbel. We selected these regions because they reflect the country's diversity in terms of wealth and modern contraceptive prevalence rates, and for logistical reasons. While Dakar has the highest proportion of women in union using a modern method (35%), Diourbel has one of the lowest (18%) (14). In terms of wealth, it is highly concentrated in Dakar where nearly the entire population is in the top 3 wealth quintiles compared to fairly even distributions across wealth quintiles in Thiès and Diourbel and 80% of the population in Kaffrine being in the lowest 2 quintiles (14). These regions also provided a mix of urban and rural settings and service delivery channels. In this study, we included three channels: static brick and mortar public sector and social franchise clinics and mobile outreach clinics that bring reproductive health services to clients (additional details below). Dioubel and Kaffrine had public sector and mobile outreach clinics only, while Dakar and Thiès also offered services from social franchise clinics.

For the quantitative component, we interviewed LARC acceptors at three service delivery channels (public sector, social franchise, and mobile outreach clinics), and re-interviewed them at four intervals (1, 3, 6, and 12 months) over a 12-month period from February 2018 to February 2019. Women who reported discontinuation of their LARC method were not contacted after the interview during which they reported discontinuing. The qualitative component included formative cognitive interviews and in-depth interviews (IDIs). We conducted cognitive interviews with current and former LARC users who were not part of the study sample prior to participant recruitment (December 2017–January 2018). Through three rounds of asking questions and using probing techniques to get women to relay their understanding of the question, words within the question, and the reason(s) for their response(s), the cognitive interviews helped refine the quantitative and qualitative instruments. We also conducted in-depth qualitative interviews in August 2018 with a sample of women who had experienced side effects at the 1- and/or 3-month follow-up interviews. This sample represented women who had experienced side effects and continued using the same method, switched methods, or discontinued.

### Data Collection Instruments

FHI 360 and MSI staff collaboratively developed baseline and follow-up quantitative surveys. In addition to demographic characteristics and contraceptive history, the baseline survey documented the LARC method initially chosen, the reason it was selected, any concerns about using the method, whether the client was told about advantages, disadvantages and side effects associated with the method, what she would do if she experienced side effects and under what circumstances she might make the decision to remove her LARC method early.

The follow-up survey instrument differed from the baseline in that it focused on side effects and other problems women experienced since the last interview, including the worst side effects and what they did to manage them. At each time point, we documented if women continued, switched, or discontinued the method they reported using from the previous survey.

An expert in qualitative methodology developed the midterm IDI instrument to investigate more extensively women's experiences with side effects. She formulated probing questions about bleeding abnormalities and other side effects, partner and social support, as well as provider counseling and treatment practices for method continuers, switchers and discontinuers who reported experiencing side effects during the first 3 months of use. The instrument also solicited women's opinions on the type(s) of information that potential LARC clients should be given before deciding to use an IUD or implant and which services would assist women who experience side effects.

### Service Delivery Point Selection

Selected service delivery points (SDPs) represented 3 of the 4 contexts where women might obtain LARCs in Senegal: public sector clinics located in both urban and rural areas; mobile outreach clinics in both urban and rural areas; and private MSI social franchise clinics in urban, peri-urban and some rural areas. Private, for-profit facilities also exist in Senegal but were not included in the sample. Mobile outreach clinics involve teams of community outreach specialists and nurses and/or nurse-midwives who travel to remote areas to provide counseling and a range of family planning methods with the intent of improving access to quality family planning services. Service days are organized in advance with community leaders to promote awareness and acceptance of the campaign.

Mobile outreach teams travel as a unit from one location to another, following a pre-arranged schedule of stops. We selected one outreach team per region and followed them to different locations on their routes. Marie Stopes International developed the sampling frame for the universe of social franchise clinics in Dakar and Thiès and regional government officials provided the names of all public sector clinics in the four study regions. We randomly selected public sector and social franchise clinics using a simple random sampling approach stratified by SDP type. Initially, we randomly selected 26 static SDPs from the four regions (13 public, 13 social franchise) with moderate to high LARC client load to expedite participant recruitment (monthly average of 20+ for public, 15+ for social franchise clinics defined by FHI 360 and MSI staff in consultation with Ministry of Health officials). However, we excluded one social franchise clinic that was unable to recruit a sufficient number of LARC clients. Similarly, another social franchise clinic was removed from the study sample, as it had gone out of business. To preserve random selection, neither clinic was replaced. Lastly, one social franchise clinic participated only through its associated mobile clinics. Thus, the final sample included 13 public clinics, 10 social franchise clinics, and 21 different stops made by mobile outreach teams, which were frequently tied to either a public sector or social franchise clinic.

### Sample Size Calculation

We calculated the desired sample size of LARC clients based on the primary outcome of method discontinuation due to side effects. Using logistic regression modeling, a sample size of 1,445 LARC acceptors was deemed sufficient to detect a 6% difference in the rate of discontinuation due to side effects between service delivery channels. We planned to enroll 1,700 LARC acceptors to allow for up to 15% attrition. Except for service channel, where we allocated sample size uniformly, sampling was not designed to balance all factors of interest, thus we accounted for some imbalance (e.g., 1/3 of sample is from rural vs. 2/3 from urban areas) in our power analyses. Since the primary analysis was a multi-variable regression model, we accounted for a level of collinearity among covariates to be included in the model (VIF = 1.82, *R*^2^ = 0.45) in the sample size calculations (15). With the proposed sample size, the power for detecting an adjusted association between a given factor and the primary outcome was 80% for two-sided comparisons with a 5% significance level.

We aimed to achieve a sample size of 567 for each service channel. We maintained a uniform recruitment period across the static clinics to allow the number of clients recruited per clinic to vary according to their volume. Mobile teams recruited LARC participants more quickly than static clinics, and their sample sizes were also based on targets for each team.

### Study Participants

With the help of healthcare providers at service delivery points, we recruited women of reproductive age (18–49 years old) and emancipated minors (15–17 years old and married) who voluntarily chose an implant (single-rod Implanon NXT) or a copper-T IUD (TCU 380A) at the three service channels, regardless of past contraceptive history. Interval IUD and implant insertions were performed before clinic providers informed eligible participants about the study and identified the research assistant (RA) onsite who could provide more information.

Recruitment focused on women with phones to facilitate follow-up data collection, but we also included women who reported having no access to phones to obtain a more inclusive and representative sample of LARC users. Our source at the time, the 2015 Senegal DHS reported that 12% of women in Senegal lacked access to a telephone. We allowed women to choose if they preferred to be contacted by phone or in person, but for logistical reasons we established a cap of 15% for the sample of women who did not have access to a phone or preferred to be interviewed face to face. Participants were compensated ~87 cents (500 CFA Franc) per interview in airtime for those interviewed by phone and $3.12 (1,800 CFA Franc) per interview for transport for women interviewed face to face.

### Data Collection

We conducted pilot tests of the baseline and follow-up interview instruments as part of data collector training and to further refine the instruments before going into the field. Trained RAs recruited and obtained informed consent from participants to conduct a baseline interview after insertion of their LARC method and to be re-contacted for follow-up interviews. Participants were also informed of the possibility of being selected for an IDI at the 6-month follow-up.

At each follow-up round, for a maximum of four rounds, we contacted participants in the manner they requested during the informed consent process until they reported discontinuation of their LARC method at a previous round or informed RAs that they no longer wanted to participate in the study. Data collectors attempted to call each phone participant up to 20 times each round (this includes re-trying the same number if the phone was turned off or out of range). The study team also drew upon the assistance of MSI community health workers (CHWs), given the need to maintain contact with study participants throughout the 12-month study period. These CHWs were affiliated with the study sites and lived in the same communities as the participants who agreed that CHWS could contact them to remind them of upcoming interviews. Women who could not be reached after attempts by both RAs and CHWs were considered missed for that round of data collection, but missing a round of data collection did not preclude attempts to reach the participant for subsequent interview rounds.

We began administration of the baseline interviews on February 6th, 2018. Recruitment at social franchise clinics was much slower than anticipated due to a miscommunication on the days of utilization of these services (used mostly on weekends when data collectors were not working). We initiated the 1-month (April 19th to May 25th, 2018) and 3-month (May 30th to June 30th, 2018) follow-ups later than scheduled due to the 1-month extension of the baseline recruitment period and the challenge of finding participants. The six-month follow-up commenced on August 1st and was followed shortly by the mid-term IDIs initiated on August 8th, 2018 (more details below). We conducted the 12-month follow-up interviews between February and March 2019.

### Data Analyses

We conducted descriptive analyses of the baseline and follow-up interviews to provide details on LARC users' demographic characteristics (i.e., age, parity, marital status, education, religion, participant/partner occupation); method selected at baseline and type of service channel where LARC methods were obtained. These variables also served as covariates in regression analyses in addition to: reasons that would prompt her to discontinue the method (husband's dislike, wanting to get pregnant, or side effects); prior use of an IUD, implant, or injectable; counseling received at baseline (on any side effect, menstrual changes, and pain); being told at baseline that her method could be removed early; and husband's knowledge of contraceptive use. We conducted bivariate analyses with these variables to identify factors associated with LARC method discontinuation at 12 months. We used chi-square tests or Fisher's exact tests for categorical factors and t-tests for continuous factors. Any factor significantly associated with discontinuation at the 0.10 significance level was included in a multivariable regression.

We used logistic regression to identify factors associated with discontinuation due to side effects by 12 months. For this analysis, we only included participants with complete 12-month follow-up outcome data and we excluded women who were lost to follow-up or discontinued due to reasons other than side effects (*n* = 960). We also conducted a separate logistic regression analysis to identify factors associated with discontinuation due to any reason by 12 months (*n* = 1,008, this includes women who discontinued for any cause as well as those with unknown cause for discontinuation). In both regressions, we used backward selection and retained only those factors significantly associated with the outcome in the final model, using a 10% significance level for removal. We included service channel regardless of significance as a control variable in the models, as sampling was stratified by this variable.

Secondly, we estimated 12-month discontinuation rates due to side effects using a cumulative incidence function (CIF) approach for competing risk analysis (16). With this approach, participants with incomplete follow up data are included. This approach accounts for the time to discontinuation due to side effects, discontinuation due to other reasons, and loss to follow up throughout the 12-month period. In competing risk analysis, a subject can experience one of a set of different events. We considered discontinuation due to other reasons a competing event whose occurrence precludes the occurrence of a discontinuation due to side effects and compared groups using Gray's test (17). We calculated time contributed to analysis in weeks from the date of method insertion at baseline to the date women reported method removal, or to the last interview date before a participant was lost to follow up or had completed 12 months of follow up without reporting a removal. We used the same CIF approach for estimating all-cause discontinuation rates, but with no competing risks. We provided discontinuation rates with odds ratios and 95% confidence intervals for discontinuation due to side effects as well as for the overall discontinuation outcome. The CIF models were unadjusted.

Statistical analyses were conducted using SAS Enterprise Guide software, version 7.15 (copyright ^©^2017 SAS Institute Inc., Cary, NC, USA).

### Qualitative Component

Shortly after initiation of the 6-month follow-up interviews, we conducted IDIs with 18 participants selected from a randomly ordered list of 550 women across all regions who were interviewed via phone and reported experiencing side effects during their 1- and/or 3-month follow-up interviews. We stratified participants by those who: (1) continued using their LARC method (continuers), (2) discontinued their method but switched to another contraceptive method (switchers), or (3) discontinued family planning altogether (discontinuers). Additionally, they were stratified by the channel where they initially obtained their LARC method: public sector or social franchise. Mobile outreach clients were not recruited for IDIs due to logistical constraints and the local belief that these clients would be fairly similar to public sector clients, because they, unlike their social franchise counterparts would more likely be disadvantaged economically and live in underserved rural areas. Resource constraints also limited the number of IDIs we were able to conduct with continuers, switchers and discontinuers in public and social franchise clinics, preventing us from reaching saturation.

We interviewed a total of 7 continuers (4 public, 3 social franchise), 5 switchers (2 public, 3 social franchise) and 6 discontinuers (all public). All mid-term IDIs were conducted face-to-face and were audio recorded with participants' permissions. Interviews were transcribed by CEFOREP staff. A consultant analyzed the IDIs in French using thematic analysis. Descriptive codes derived from research questions and the interview guide were applied to the data and additional codes were created from emergent themes based on an inductive process. The coding of qualitative data followed a manual process based on a careful reading and re-reading of each transcript followed by annotation of the appropriate theme associated with each narrative segment of the interview, revealing trends in the attitudes, practices, and experiences of participants.

FHI 360's Protection of Human Subjects Committee (Durham, NC, USA), MSI's Ethics Review Committee (London, UK), and the Comité National d'Ethique pour la Recherche en Santé in Senegal reviewed and approved the protocol for this study. All study staff completed training on research ethics, the study protocol, and informed consent administration.

## Results

**Figure 1:**
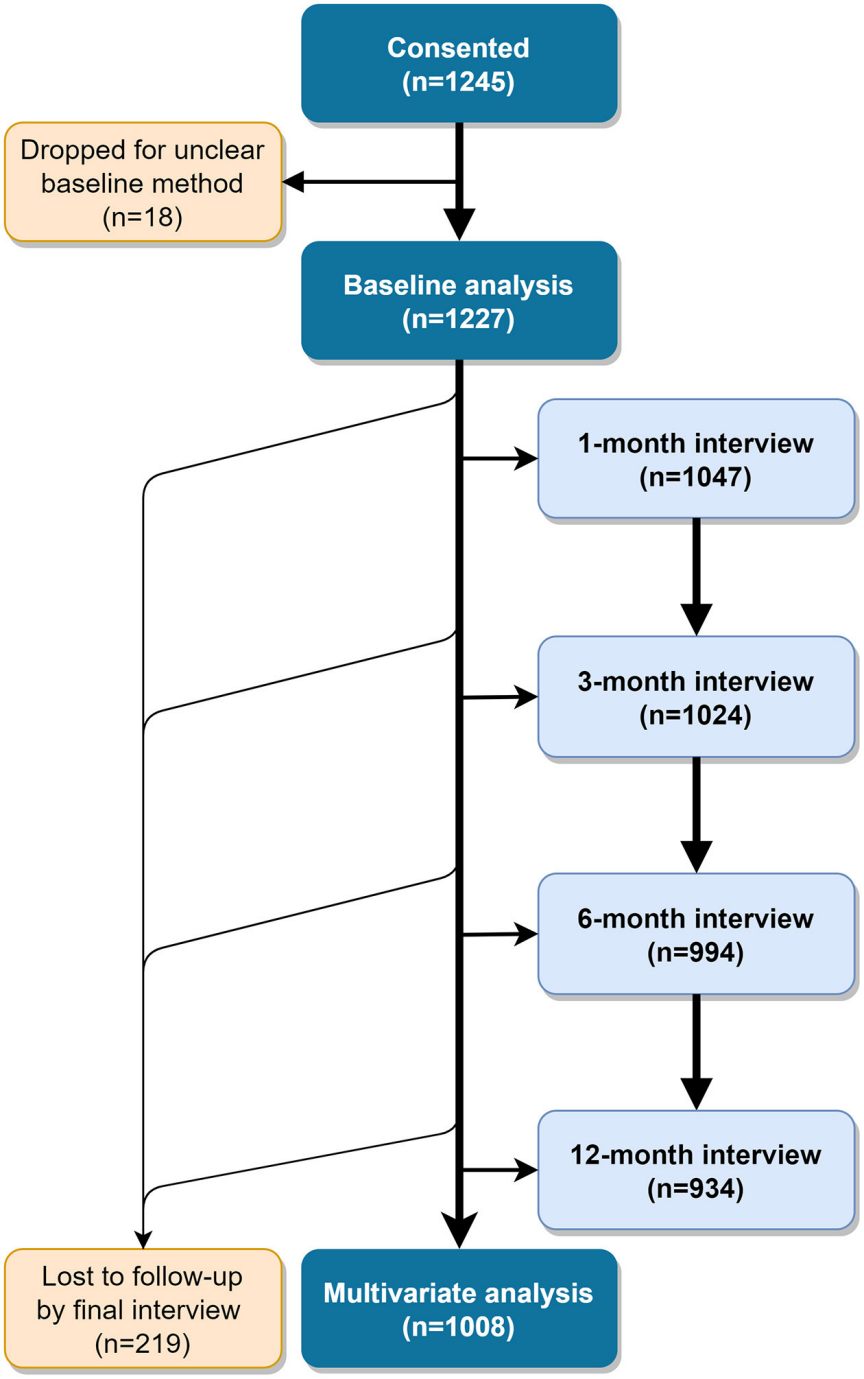
Participant flow diagram.

We enrolled 1,245 women who chose either an IUD or an implant by April 6th, 2018. For 18 of these women, the method selected at baseline was unclear and they were dropped from analyses, for a total analysis population of 1,227 (Figure 1). Eighteen percent of these did not have access to a phone or preferred to be interviewed in person, exceeding our intended cap of 15%.

Table 1 presents participant characteristics by service channel. Participants were, on average, 28 years old with three children, and nearly all were married and Muslim. About half had not completed primary school and were not employed. Most participants (54%) were recruited from Dakar, as SDPs in that region were more likely to meet our criterion for moderate to heavy client load. At baseline, 28% of women selected the IUD, while the remaining 72% chose an implant. About 36% were recruited from public sector, 15% from social franchise and 49% from mobile outreach clinics. Mobile outreach clients tended to differ from public sector and social franchise clients as they were located mostly in rural areas, had more children, were less educated and a greater proportion of them chose the IUD. Thus, mobile outreach and public sector clients were not as similar as we expected. We subsequently discovered that participating study clients served by mobile outreach services came from particularly disadvantaged areas along outreach routes.

**Table 1:** Baseline client characteristics, by recruitment service delivery point.

**Characteristic**	**Total**	**Public**	**Mobile clinic**	**Social franchise**
	**(*n* = 1,227)**	**(*n* = 442)**	**(*n* = 607)**	**(*n* = 178)**
Mean age (years)	28.3	27.8	28.4	29.3
Mean number of children	3.2	2.8	3.6	2.9
Married	97.6%	97.3%	98.7%	94.9%
Muslim	98.0%	95.9%	99.5%	97.8%
Employed	44.4%	44.0%	44.0%	46.6%
Urbanicity				
Rural	22.6%	2.5%	43.8%	0%
Peri-urban	14.8%	9.7%	16.6%	21.3%
Urban	62.6%	87.8%	39.5%	78.7%
Highest level of education				
None	46.4%	34.9%	57.8%	36.4%
Primary	28.9%	32.9%	25.7%	30.1%
Secondary	19.9%	23.4%	14.2%	30.7%
More	4.7%	8.8%	2.3%	2.8%
Method selected at baseline				
IUD	27.5%	21.3%	33.6%	21.9%
Implant	72.5%	78.7%	66.4%	78.1%
Region				
Dakar	54.2%	82.6%	29.0%	69.7%
Diourbel	14.7%	9.5%	22.7%	0%
Kaffrine	14.2%	6.1%	24.2%	0%
Thiès	17.0%	1.8%	24.1%	30.3%

A slightly greater proportion of women interviewed in person—vs. via telephone—were from rural areas (26% compared to 22%), had no formal education (58 vs. 44%) and resided in Dakar (62 vs. 53%) or Kaffrine (20 vs. 13%). Phone and in-person populations were similar on other characteristics (data not shown). Two hundred nineteen women (17.8%) did not complete 12 month follow up outcome data. Women lost to follow-up were more likely to be younger and to have initially selected the implant than women who completed the study and were not significantly different on other demographic characteristics (data not shown).

### Discontinuation

The analysis of factors associated with discontinuation at 12 months due to side effects included 960 women who had complete follow up outcome data (this excludes discontinuations due to reasons other than side effects); among these, 7.5% discontinued due to side effects by 12 months. For the analysis of associations with discontinuation due to any reason, we included 1,008 women with complete follow up data for this outcome. Among these, 12% had discontinued the method chosen at baseline by 12 months.

In the analysis of discontinuation due to side effects, the method chosen at baseline and parity were found to be significant at the 0.10 level and were included in the multivariable model (Table 2). For all-cause discontinuation, the results were the same: That is, in both models baseline method and parity remained after backwards selection, indicating that women who chose the IUD at baseline were more likely to discontinue than those who chose the implant, and the likelihood of discontinuation decreased for women with more children. Regardless of significance, we kept service channel in the models as recruitment was stratified by channel. However, service channel did not emerge as a significant factor in discontinuation due to side effects or for any reason (Table 3).

**Table 2:** Bivariate associations between categorical variables of interest and discontinuation of baseline method by 12-months (due to side effects and all-cause).

	***N* for row**	**Discontinued due to side effects**		***p***	***N* for row**	**Discontinued (all-cause)**		***p***
		**#**	**%**			**#**	**%**	
**Region**								
Dakar	527	37	7.0%	0.64	548	58	10.6%	0.45
Thiès	150	15	10.0%		159	24	15.1%	
Diourbel	123	8	6.5%		131	16	12.2%	
Kaffrine	160	12	7.5%		170	22	12.9%	
**Baseline LARC method**								
IUD	273	35	12.8%	**<0.0001**	290	52	17.9%	**0.0002**
Implant	687	37	5.4%		718	68	9.5%	
**Religion**								
Muslim	942	69	7.3%	0.14	989	116	11.7%	0.21
Christian	18	3	16.7%		19	4	21.1%	
**Urbanicity**								
Rural	210	13	6.2%	0.72	223	26	11.7%	0.71
Peri-urban	141	11	7.8%		151	21	13.9%	
Urban	609	48	7.9%		634	73	11.5%	
**Highest level of education**								
None	426	30	7.0%	0.14	454	58	12.8%	0.70
Primary	276	16	5.8%		293	33	11.3%	
Secondary	199	18	9.0%		201	20	10.0%	
More	57	8	14.0%		57	8	14.0%	
**Married**	938	72	7.7%	0.19	984	118	12.0%	0.63
**Prior use hormonal contraception**	589	40	6.8%	0.35	620	71	11.5%	0.63
**Employed**	433	27	6.2%	0.18	455	49	10.8%	0.32
**Partner employed**	909	70	7.7%	0.50	954	115	12.1%	0.59
**Access to a phone**	930	70	7.5%	0.86	976	116	11.9%	0.92
**Side effects would make her want to remove LARC early**	529	46	8.7%	0.13	557	74	13.3%	0.14
**At baseline, counseled on**								
Side effects	616	49	8.0%	0.50	647	80	12.4%	0.58
Menstrual changes	531	42	7.9%	0.61	556	67	12.1%	0.90
Pain (abdominal, headache, other)	321	29	9.0%	0.21	334	42	12.6%	0.66
Ability to remove IUD/implant early	825	60	7.3%	0.46	866	101	11.7%	0.50
**Social support**								
Decided to use IUD/implant on her own	718	57	7.9%	0.40	753	92	12.2%	0.64
Partner knows using IUD/implant	651	50	7.7%	0.79	680	79	11.6%	0.65

**Table 3:** Adjusted OR from final model for factors associated with discontinuation by 12 months due to side effects and all-cause discontinuation.

	**Discontinuation by 12 months due to side effects**		**Discontinuation by 12 months for any reason**	
	**(*****n*** **=** **960)**		**(*****n*** **=** **1,008)**	
	**OR**	**95% CI**	**OR**	**95% CI**
Mobile outreach vs. public sector clinic	0.77	(0.45, 1.33)	0.93	(0.61, 1.44)
Social franchise vs. public sector clinic	0.86	(0.41, 1.83)	1.03	(0.57, 1.86)
IUD vs. implant	3.15	(1.91, 5.22)^***^	2.39	(1.60, 3.58)^***^
Parity	0.81	(0.70, 0.94)^**^	0.86	(0.77, 0.96)^**^

** *p < 0.01;*

*** *p < 0.0001*.

As expected, the predominant reason for all-cause discontinuation was side effects. Other reasons reported for discontinuation included the disapproval of others, wanting to or becoming pregnant, relationship changes, and other health problems (Table 4).

**Table 4:** Percentage of women giving each reason for all-cause discontinuation, by method (*N* = 120).

**Reason**	**IUD**	**Implant**
	**(*n* = 52) %**	**(*n* = 68) %**
Did not like side effects	67.3	54.4
Husband disapproves	9.6	20.6
Want to get pregnant	7.7	22.1
Expulsion	7.7	0
Serious health problems	5.8	13.2
Relationship change	5.8	8.8
Belief that method is no longer working	5.8	1.5
Became pregnant	5.8	1.5
In-laws disapprove	1.9	0
Other (other family members' disapproval, resting for 6 months, nearing menopause, presence of cysts)	7.7	1.5

*Multiple responses were possible*.

Given that one of the main factors associated with discontinuation was the choice of method, we examined differences in characteristics between women who chose the IUD or an implant at baseline. As Table 5 shows, there are some significant differences between IUD and implant users. IUD users on average tended to be a little older, have more children, were more likely to live in a rural area, and were less likely to have already tried hormonal contraception than implant users. While most IUD and implant users stated at baseline that side effects would make them remove their method early and reported that their partners were aware of their LARC use, IUD users were more likely than implant users to say they would remove their LARC method early for that reason and implant users were more likely than IUD users to report that their partners were aware of their method use. Implant users were also less likely to be counseled on pain as a side effect. Whether implant or IUD user, we recruited most of our participants from urban facilities.

**Table 5:** Bivariate associations between variables of interest and method accepted at baseline.

	**#**	**IUD**	**#**	**Implant**	***p***
		**(*n* = 337)**		**(*n* = 889)**	
**Mean age**	337	30.7	890	27.4	**<0.0001**
**Mean number of children**	337	3.9	890	2.9	**<0.0001**
**Region**					
Dakar	150	44.5%	515	57.9%	**<0.0001**
Thies	88	26.1%	92	10.3%	
Diourbel	37	11.0%	137	15.4%	
Kaffrine	62	18.4%	146	16.4%	
**Religion**					
Muslim	331	98.2%	871	97.9%	0.69
Christian	6	1.8%	19	2.1%	
**Urbanicity**					
Rural	94	27.9%	183	20.6%	**0.003**
Peri-urban	58	17.2%	124	13.9%	
Urban	185	54.9%	583	65.5%	
**Highest level of education**					
None	160	47.5%	408	46.1%	0.83
Primary	92	27.3%	262	29.6%	
Secondary	67	19.9%	176	19.9%	
More	18	5.3%	40	4.4%	
**Married**	332	98.5%	865	97.3%	0.21
**Prior use hormonal contraception**	94	28.1%	390	43.9%	**<0.0001**
**Employed**	162	48.1%	382	43.0%	0.11
**Partner employed**	320	96.4%	842	95.5%	0.48
**Access to a phone**	327	97.0%	860	96.6%	0.72
**Side effects would make her want to remove LARC early**	199	59.1%	469	52.9%	0.05
**At baseline, counseled on**					
Any side effects	209	62.0%	571	64.4%	0.44
Menstrual changes	173	51.3%	503	56.7%	0.09
Pain (abdominal, headache, other)	128	38.0%	260	29.3%	**0.004**
Ability to remove IUD/implant early	297	88.1%	748	84.3%	0.09
**Social support**					
Decided to use IUD/implant on her own	258	76.6%	653	73.6%	0.29
Partner knows using IUD/implant	199	59.1%	606	68.3%	**0.002**

**Figure 2:**
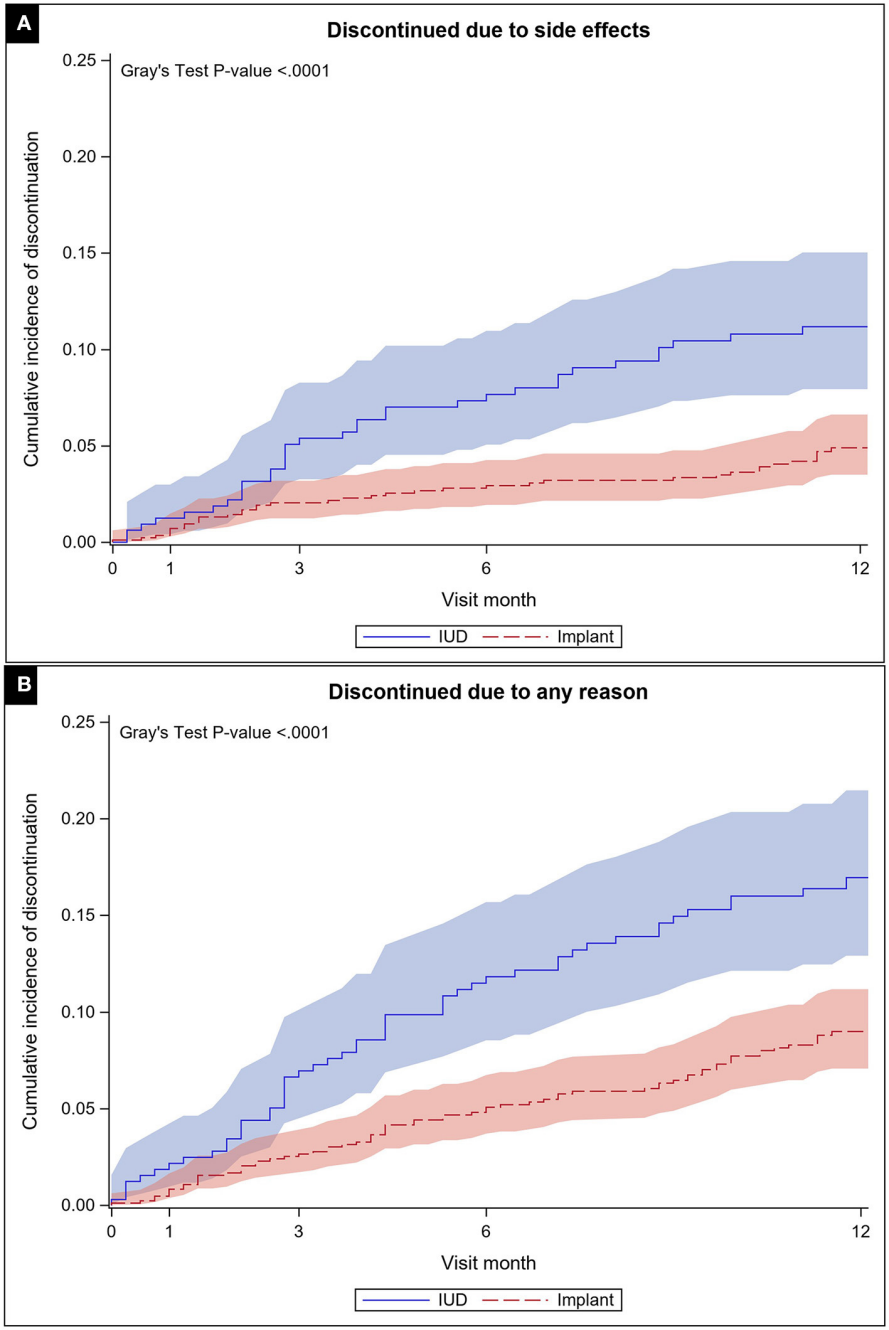
Cumulative incidence function with confidence limits for discontinuation due to side effects **(A)** and all-cause **(B)** through 12 months of follow up by method chosen at baseline.

We also estimated the discontinuation rates by method. Based on the cumulative incidence function, the 12-month discontinuation rate due to side effects for IUDs was 11.2% (95% CI: 7.9–15.0%) and for implants was 4.9% (95% CI: 3.5%-6.6%). For all-cause discontinuation, the 12-month discontinuation rate for IUDs was 17.0% (95% CI: 12.9–21.5%) and for implants was 9.0% (95% CI: 7.1–11.2%). Figure 2 presents results of the cumulative incidence functions for discontinuation due to side effects for each method (*p* < 0.0001) and for all-cause discontinuation (*p* < 0.0001). For these analyses, we used the sample of *N* = 1,227 with recorded baseline method. Participants lost to follow-up contributed to the rate estimation up to the time they were lost.

### Side Effects

Overwhelmingly, the most frequently cited reason for discontinuation, among both IUD and implant users, was dislike of side effects (Table 4). While no other reason was very common for IUD users, one-fifth of those discontinuing an implant mentioned a desire to become pregnant and another fifth mentioned husband's disapproval. Three IUD users and one implant user reported becoming pregnant while using the method.

At baseline, women reported several side effects they thought might prompt them to remove the IUD/implant early. The most frequently mentioned side effects in order of magnitude were change to menses, abdominal pain, change in the quality of sex, headaches, and weight change (data not shown). Many of these were also the most commonly experienced by both IUD and implant users, with change to menses being the most frequent (reported by 36% of IUD users at baseline and decreasing each interview round to 12% of users at 12-months; reported by a quarter of implant users at every round). At every survey round, women reported change to menses as the worst side effect (34–50% of IUD users and 50–60% of implant users). In IDIs, participants described bleeding for twice as long as normal, or having irregular periods that made them consider removing or actually remove the method. Excess bleeding caused concern.

“*…The bleeding came almost all the time… so…I removed the implant even before the follow-up at three months. I removed it to use the IUD and it [the IUD] was worse – I was bleeding every day. When the IUD lasted a week I went back to see the midwife to tell her that I was still bleeding and I removed it.”* ***Discontinuer, Implant and IUD, Dakar, age 37***

“*Sometimes the flow is abundant, sometimes the flow is irregular. … A month ago I thought I would go to the hospital to explain to the midwife that I feel that it is making me bleed… but finally I resigned myself and did not go. The bleeding stopped and I noticed that every month it's the same thing.”* ***Continuer, Implant, Dakar, age 37***

In the qualitative interviews, abdominal pain and change to the quality of sex were the other most frequently reported side effects for IUD users and the ones women most frequently thought of as the worst. For implant users, abdominal pain and headaches were the next most common, after change to menses, with menstrual changes overwhelmingly reported by women to be the worst side effect of all. Respondents in IDIs mentioned that the change to quality of sex was because their husbands could feel the IUD strings during intercourse.

“*…With the IUD, at the time of intercourse, the wire was bothering him. I went back to the midwife who told me it would pass…. But my husband told me that at the time of intercourse the wire embarrassed him… The side effects affected our relationship because we did not have sex as he wanted.”* ***Switcher, IUD to implant, Dakar, age 25***

Several IUD discontinuers noted their husbands were “pricked” by the IUD during sex; although two of these had expulsions, neither had an IUD reinserted. For implant users, changes in bleeding patterns caused problems with spouses, since many women noted that they do not have sex during menstruation. As one who discontinued after 1.5 months explained, “It's not fair [to the husband] if the wife bleeds all the time.” Another participant who switched was given money by her husband to return to the clinic because “bleeding all the time is not normal”; another shared, “He told me to return to the midwife [about the bleeding].” On the other hand, women whose menses disappeared also stopped using a method; one husband asked his wife to return to the midwife because “it's not normal not to have menses.”

At baseline, two-thirds of women reported being counseled on the potential side effects of their chosen method; this did not vary by method type (Table 5). While some participants in IDIs mentioned being counseled in a way that reassured them when side effects appeared, others suggested that counseling on side effects was poor or non-existent and would have been appreciated by clients.

“*Providers should tell us everything about the methods and not hide anything from FP users or wait for us to return with this or that side effect. Whatever method they desire to give us, they must first tell us about everything.”* ***Continuer, Implant, Dakar, age 25***

“*I have no problem with bleeding a little. It lasts 3 or 4 days. It was not a problem for me because the midwife had reassured me about the possible side effects. She told me it's normal… and to come back in case of side effects.”* ***Continuer, Implant, Dakar, age 37***

“*The provider had told me about the weight gain and the late periods. She had explained to me the possible side effects so when I saw these side effects I went back to see the midwife. When I told her that I was going to remove it, she agreed, but she told me to wait for up to 6 months [because] maybe these effects could be normal again. That did not suit me because of the weight gain. I finally removed it.”* ***Switcher, Implant to pills, Dakar, age 20***

Over the course of the study, about half (48.6%) of IUD users and a third (35.2%) of implant users who experienced a side effect reported asking a provider for help managing it. Remaining participants said they talked to friends, family or dealt with it on their own. In IDIs, most women who experienced particularly intense side effects (e.g., bleeding for more days than normal or much heavier than anticipated) sought help from midwives, who often gave the women medication to stop the bleeding. Many of the discontinuers were women for whom the medication did not work or worked only for a day or two. One continuer who did not have money or time to return to a midwife noticed that the bleeding was the same month after month, talked to a friend about it, and “resigned herself” to having extra days of menses while she used the implant.

## Discussion

At 12 months, women in this study who chose an IUD were more likely to stop using that method than implant users. Twelve-month discontinuation was also strongly associated with parity, suggesting that the more children a woman has, the more motivated she is to continue contraception and avoid additional pregnancies. As expected, side effects were reported as the predominant reason for all-cause discontinuation. We were not able to detect an association between 12-month discontinuation and baseline counseling, social support indicators, or other demographic characteristics, nor did we find evidence that discontinuation varied by service channel.

For IUD users in this study, the 1-year rate of discontinuation due to side effects was markedly higher than reported in the 2017 Senegal DHS (11% compared to an un-weighted rate of 4% in the DHS) (18). Given the challenges we experienced with recruiting the desired sample size and loss-to-follow-up, the higher discontinuation rate may be an artifact of our study. For all-cause discontinuation of IUDs, we found a 17% 12-month discontinuation rate as compared to 8% in the 2017 DHS (18). For implants, however, our results are similar to the DHS both for discontinuation due to side effects (4.9 vs. 4.8% in the DHS) and all-cause (9 vs. 11% in the DHS) (14). In other settings, others have reported discontinuation rates higher than our study's: O'Neil-Callahan et al. (8) reported a 14.9% 12-month discontinuation for copper IUD users and 16.6% for implant users; Peipert et.al. (19) found a 16% discontinuation rate for copper IUD and 17% for implants. However, some of the differences observed between reported rates may be due to methodological differences in the estimation of the discontinuation rates. We used a rigorous approach that uses all available data and accounts for competing risks when looking at cause-specific discontinuation (16). Although approaches accounting for the time to discontinuation for different causes is clearly preferred by some, researchers use different methods that are not always clearly described in the literature (16).

Our in-depth interviews support the importance of side effects in a woman's LARC experience and complement our longitudinal quantitative data, which indicate that parity and method selection were important predictors of method continuation. Nevertheless, there is no doubt that side effects are a major contributor to discontinuation. Since our study did not have a good measure of severity of each side effect and how that changed over time, future studies should consider the timing and severity of reported side effects in an attempt to better predict women at risk of discontinuation and provide better counseling and side effect management techniques. Helping women understand the potential side effects of contraceptive choices and prepare for how that might impact their lifestyle may not reduce discontinuation or switching rates, but it may reduce levels of anxiety associated with side effects. In the era of increasing self-care for family planning (20), and because many women turn to friends or family for help to manage side effects, finding ways to provide this information independent of health facilities is also important. One option could be via digital services, such as Nivi (https://www.nivi.io/), which provide women with direct access to family planning information, including side effects.

Several studies have shown no association between counseling quality and continuation (11, 21–24). However, there is also evidence to the contrary, that counseling does positively affect continuation rates, such as research published in 2019 (25) based on the Method Information Index (MII)–an indicator of counseling quality–as well as studies of injectable contraception in China (26), Mexico (27), and India (28). Interventions in these success stories, though, tended to provide higher intensity counseling and follow-up than might normally occur in the field. Given this and mixed results in general, improving counseling alone may not be sufficient to reduce discontinuation. Indeed, side effects counseling did not emerge as a significant factor in our analyses. Moreover, women who reported being counseled still switched or discontinued and those who reported being poorly counseled still continued. Therefore, while effective counseling might reduce fears and concern, it is unclear that it invariably promotes continuation.

This study had several limitations. We did not achieve our intended sample size at social franchise clinics. Furthermore, 18% percent of our sample was lost to follow-up by the 12th month. Although not a small number, it is not unexpected for this type of longitudinal study. Our sample size calculation provided for 15% loss to follow-up. However, even after extending the baseline recruitment period for an extra month, we were unable to obtain the desired sample size to offset this loss. There is always the potential for biases with incomplete data if those lost to follow-up are more or less likely to discontinue or have a different discontinuation profile. Our methods assumed that data are missing at random and all data available are used in the different analyses without making additional assumptions.

While we selected SDPs to include a population that is similar in wealth, contraceptive prevalence, and urbanicity to that of the DHS population in the 4 regions of interest, the restriction of the sample to larger clinics, the convenience-based sampling of participants, and the unknown population size of those served by mobile outreach affected our ability to formally construct sampling weights. Further, the population in the catchment areas of mobile outreach clinics overlapped with those of other service channels.

This prospective study confirmed that, in Senegal, LARC discontinuation is low, and that parity and method selection predict continuation, while side effects remain a major concern for women and drive their decisions to discontinue the IUD and implant. Furthermore, women using these methods continue to complain of side effects up through 12 months, but only half seek assistance from a health provider. Better communication between providers and clients about these side effects, particularly at the time of method initiation is important as intimated by women interviewed in-depth in our study. Indeed, a recent study in Senegal found that counseling on side effects was poor (only 45% of LARC clients counseled) (29). Tools to address common side effects, like the NORMAL counseling tool for menstrual bleeding changes (30) exist and could be incorporated into provider training and counseling materials for clients. With menstrual changes being a major side effect and reason for discontinuation, focus on improving counseling on this side effect would be valuable. As family planning grows in popularity, in Senegal and elsewhere, we must ensure that women are offered their free choice from a full range of high-quality methods, including LARCs, and that service provision includes comprehensive counseling about side effects.

## Data Availability Statement

The datasets presented in this study can be found in online repositories. The names of the repository/repositories and accession number(s) can be found below: De-identified data from our study is now publicly accessible via the Harvard Dataverse: https://dataverse.harvard.edu/dataverse/LARCsenegal.

## Ethics Statement

The studies involving human participants were reviewed and approved by (1) Protection of Human Subjects Committee at FHI 360 (Durham, NC, USA), (2) MSI's Ethics Review Committee (London, UK), (3) Comité National d'Ethique pour la Recherche en Santé in Senegal. The patients/participants provided their written informed consent to participate in this study.

## Author Contributions

DC-Q contributed to study conceptualization, design development, implementation, data interpretation, manuscript development, and review. MD contributed to study implementation, data interpretation, manuscript development, and review. ME contributed to study implementation, data entry, management, analysis, verification and interpretation, manuscript development, and review. AY contributed to study implementation, data entry, management, manuscript development, and review. MC contributed to study design development, data analysis, verification, interpretation, manuscript development, and review. AB contributed to data analysis, verification and interpretation, manuscript development, and review. JS contributed to study conceptualization, design development and implementation, manuscript development and review. All authors contributed to the article and approved the submitted version.

## Funding

This study was made possible by the generous support of the American people through the U.S. Agency for International Development (USAID), provided to FHI 360 through cooperative agreement numbers AID-OAA-A-15-00045 and AID-OAA-A-14-00036.

## Author Disclaimer

The contents are the responsibility of FHI 360 and do not necessarily reflect the views of USAID or the United States Government.

## Conflict of Interest

The authors declare that the research was conducted in the absence of any commercial or financial relationships that could be construed as a potential conflict of interest.

## Publisher's Note

All claims expressed in this article are solely those of the authors and do not necessarily represent those of their affiliated organizations, or those of the publisher, the editors and the reviewers. Any product that may be evaluated in this article, or claim that may be made by its manufacturer, is not guaranteed or endorsed by the publisher.
